# Evaluation of Propylene Glycol and Essential Oil Supplementation on Growth Performance, Feed Efficiency, Serum Biochemical Indices, Hematological Parameters, and the Expression of Antifreeze IV and Lipid Metabolism-Related Genes in Nile Tilapia

**DOI:** 10.3390/ani16040615

**Published:** 2026-02-14

**Authors:** Doaa R. Saleh, Abeer F. El-Nahas, Walaa S. H. Abd El Naby, Hadir A. Aly, Ehab El-Haroun, Shymaa A. Khatab

**Affiliations:** 1 Department of Animal Husbandry and Animal Wealth Development-Genetic Laboratory, Faculty of Veterinary Medicine, Alexandria University, Alexandria 21561, Egypt; vet7.doaaali@alexu.edu.eg (D.R.S.); walaa.hamouda@alexu.edu.eg (W.S.H.A.E.N.); shymaa.khattab@alexu.edu.eg (S.A.K.); 2 Fish Rearing Laboratory, Aquaculture Division, National Institute of Oceanography and Fisheries (NIOF), Alexandria 11516, Egypt; hadir.aly@alexu.edu.eg; 3 Department of Integrative Agriculture, College of Agriculture and Veterinary Medicine, United Arab Emirates University, Al Ain P.O. Box 15551, United Arab Emirates

**Keywords:** Nile tilapia (*Oreochromis niloticus*), propylene glycol, essential oils, *AFPIV* gene, gene expression

## Abstract

Aquaculture output, sustainability, and profitability can be enhanced by using functional feed additives. The effect of supplementation with two different dietary levels of propylene glycol (PG) and essential oils (EOs) was evaluated in Nile tilapia. A total of 150 juvenile fish were randomly allocated into five groups. Growth performance, feed utilization, serum biochemistry, hematology, and gene expression were assessed. PG supplementation significantly improved growth performance, feed conversion, protein efficiency, and energy utilization. Both additives significantly reduced cortisol and glucose levels and altered liver enzymes and lipid profiles. PG improved immunological indices, while hematological responses were dose-dependent; both EOs and PG increased hematocrit, mean corpuscular volume (*MCV*), and mean corpuscular hemoglobin (*MCH*). Moreover, the high PG dose significantly increased platelet counts, reduced hemoglobin (Hb), and elevated hematocrit. Additionally, EOs significantly upregulated antifreeze protein IV (*AFPIV*) and fat metabolism-related genes in a dose-dependent manner, indicating a potential role in lipid mobilization and stress tolerance. Expression analysis of the immunoglobulin H (*IGMH*) gene revealed a significant increase in PG-supplemented groups, suggesting its immunostimulatory potential. Overall, PG enhanced immunity and growth performance, while EOs promoted *AFPIV* and fat metabolism gene expression. Therefore, PG and EO supplementation could serve as an effective functional strategy to enhance *O. niloticus* growth, stress adaptation, and immune resilience.

## Simple Summary

The effects of propylene glycol (PG) and essential oils (EOs) as functional feed additives on growth, physiological performance, and stress resilience in Nile tilapia (*Oreochromis niloticus*) were evaluated. PG supplementation markedly improved growth performance, feed efficiency, protein utilization, and energy conversion, indicating enhanced metabolic activity. Both PG and EOs significantly reduced serum cortisol and glucose, reflecting improved stress tolerance and hepatic modulation. PG also enhanced immune indicators, while EOs exhibited dose-dependent effects on hematological parameters and lipid metabolism. Notably, EOs upregulated antifreeze protein IV (*AFPIV*) and lipid metabolism-related genes, suggesting a role in thermal adaptation and lipid mobilization. PG induced higher expression of the immunoglobulin heavy chain gene (*IGMH*), demonstrating its immunostimulatory potential. Overall, PG improved growth and immune competence, while EOs promoted molecular adaptation to stress.

## 1. Introduction

The rapid expansion of the global market for Nile tilapia (*O. niloticus*) and its remarkable adaptability to diverse environmental conditions have made it a preferred species in aquaculture production systems [1]. It is now one of the most widely farmed fish worldwide, with consumption steadily increasing due to rising demand for affordable and sustainable protein sources [2].

In intensive aquaculture, the use of feed additives has become indispensable for maintaining health, enhancing growth, and improving resilience to stressors. Among these, functional feed additives (FFAs) such as prebiotics, probiotics, and phytobiotics have received growing attention for their roles in optimizing nutrition and reducing disease outbreaks [3]. Recent findings, including those reported by Hossain [4], demonstrate that FFAs can improve feed utilization, enhance overall health and durability, and support sustainability in plant-based diets. They also offer a cost-effective and environmentally friendly alternative that reduces dependence on traditional antibiotics and high-cost fish meals.

Energy supplementation is also critical in aquaculture since fish are poikilothermic, and energy utilization varies considerably depending on farming systems, species, and water temperature [5].

Propylene glycol (PG) and essential oils (EOs) are examples of feed additives that are generally recognized as safe for animals, consumers, and the environment [6]. They have been shown to enhance appetite, modulate immune responses, exert antioxidant effects, and display antiparasitic, antibacterial, anesthetic, and antistress actions [7]. Propylene glycol (1,2-propanediol), a synthetic hydrocarbon derived from propane cracking, has been widely used in terrestrial animals as an energy source. In broilers, PG improved feed palatability and growth performance [8], while in salmonids, it enhanced body weight gain and feed conversion ratio [9]. PG is also anti-ketogenic, reducing liver triglycerides and non-esterified fatty acids and improving glucose balance [10]. In fish, PG supplementation has been associated with improved feed intake, growth, feed conversion, and serum protein levels, particularly under stress conditions [11].

Similarly, EOs, which are lipophilic mixtures of volatile secondary metabolites derived from aromatic plants, are gaining attention as eco-friendly additives in aquaculture [12]. Rich in bioactive compounds such as terpenes, phenolics, and aldehydes, EOs exhibit antibacterial, antioxidant, and immunostimulatory properties [13]. Their dietary inclusion not only reduces reliance on antibiotics but also enhances digestion, gut microbial balance, growth, and overall fish welfare [14].

Both PG and EOs also influence lipid metabolism at the molecular level. PG alters lipogenic enzyme activity and transcription factors such as diacylglycerol acyltransferase (*DGAT1*), fatty acid synthase (*FAS*), peroxisome proliferator-activated receptor gamma (*PPARγ*), carbohydrate response element-binding protein (*ChREBP*), and sterol regulatory element-binding protein (*SREBP-1c*) in ruminants [15]. In aquatic species, dietary phytobiotics such as garlic, carvacrol, and thymol have been shown to modulate lipid metabolism-related genes in *Sparus aurata*, improving both metabolic activity and protein synthesis [16]. Likewise, blends of EOs in poultry diets modulated lipid oxidation and lipogenesis genes in a dose- and tissue-specific manner [17].

Interestingly, recent studies suggest that antifreeze protein type IV (*AFPIV*), originally identified in teleosts [18,19,20], may also be linked to lipid metabolism in fish. Its expression has been detected in various tissues of *O. niloticus* under different seasonal conditions, indicating multifunctional roles beyond cold adaptation [21].

Therefore, this study aimed to evaluate the effects of dietary supplementation with different levels of PG and EOs on *O. niloticus* including growth performance, feed utilization, serum biochemical and hematological parameters, and the expression of *AFPIV*, fat metabolism-related genes, and selected immune-related genes.

## 2. Materials and Methods

### 2.1. Ethical Approval

The experiment was performed following the guidelines for fish research from the animal ethics committee at Alexandria University.

### 2.2. Fish, Diets, and Experimental Design

Monosex, all-male, Nile tilapia (*O. niloticus*) juveniles (mean weight: 57.5 ± 2.5 g; *n* = 500) were obtained from a commercial hatchery in Idko, Behera, Egypt. Nile tilapia juveniles were acclimatized for 15 days in 2 m^3^ circular fiberglass tanks with continuous aeration and a 30% daily water exchange. After the acclimatization period, 150 apparently healthy Nile tilapia juveniles were selected for this study.

Basal diet (pellet size: 1 mm) containing 30% crude protein was supplied to all fish groups at a fixed feeding rate during the adaptation period until the start of the feeding trial. The ingredients and chemical composition of the basal diet are listed in Table 1. Two commercial supplementary products were used in this study at two different concentrations each. PG (99% purity) was used at 5 and 7.5 mL/kg diet [22,23]. EOs was used at 1 and 2 mL/kg [24]. Both PG and EOs were added to the basal diet by spraying onto the prepared diet after two weeks of fish acclimatization and continued for 60 days [24].

The fish were randomly assigned to five experimental groups in triplicate (10 fish/hapa). The fish were allocated to 15 hapas, 1 m^3^/each (100 × 100 × 100 cm^3^). Five dietary treatments were conducted as follow: group 1 (G1) was kept as a control fed the basal diet without any treatment, group 2 (G2) received 5 mL/kg PG in the basal diet, group 3 (G3) received 7.5 mL/kg PG in the basal diet, group 4 (G4) received 1 mL/kg EOs in the basal diet, and group 5 (G5) received 2 mL/kg EOs in the basal diet. Fish were fed experimental diets at a daily feeding rate of 5% of their live body weight for 6 days a week allowing fish to consume any residual feed. Experimental diets were introduced by hand (as this method enables regular inspection of the fish [25] two times daily, at 9 a.m. and 2 p.m.). The feeding rate was adjusted biweekly based on changes in body weight. Dead fish were recorded daily.

### 2.3. Physicochemical Parameters of Water

Water temperature, pH, dissolved oxygen (DO), and ammonia (NH4) were measured weekly. PH, DO, and temperature were taken using a portable multiparameter meter (Lovibond®, Dortmund, Germany). Ammonia was quantified via colorimetry (8 h fixed samples) using a HANNA multiparameter spectrophotometer (HI83399, Romania).

All monitored water quality parameters remained stable throughout the trial; no significant differences were observed among the treatment groups as shown in Table 2. The temperature was maintained at 26.50–26.79 °C throughout all treatments, and the pH values (7.49–7.65) remained within the optimal range. The DO levels showed remarkable stability (4.34–4.43 mg/L). Ammonia concentrations, both as NH_4_ (0.46–0.64 mg/L) and NH_3_ (0.01 mg/L across all groups), remained well below the harmful limits for *O. niloticus*.

### 2.4. Biometric Indices Assessment

At the end of the 60-day experimental period, fish in each hapa were counted and weighed to evaluate growth performance and feed utilization parameters [26] by using the following equations: weight gain (WG, g) = 100 × (final body weight − initial body weight), daily weight gain (DWG, g) = (WG/number of days), specific growth rate (SGR/day%) = 100 × ln (final body weight) − ln (initial body weight))/test days, condition factor (K) = 100 (W/L^3^), where W = fish weight and L = fish length, feed intake (FI, g/fish/day) = feed consumption (g)/average biomass (g) × days, feed conversion ratio (FCR) = total feed consumption (gm)/total weight gain (g), protein efficiency ratio (PER, %) = 100 × (total weight gain (g)/protein intake (g)), protein productive value (PPV, %) = 100 × (protein gain (g)/protein intake (g)), and energy utilization (EU, %) = 100 energy gain (kcal)/energy intake (kcal).

### 2.5. Blood Sampling and Hematological Parameter Analysis

Two types of blood samples were collected from each fish via the caudal vein. For the whole blood, samples were collected with the anticoagulant EDTA. A separate sample was collected without an anticoagulant for serum separation.

#### 2.5.1. Hematological Parameter Analysis

Blood samples diluted with Natt and Herrick’s solution (Sigma-Aldrich, St. Louis, MO, USA) were used to determine white blood cell (WBC) and red blood cell (RBC) counts. The cyanmethemoglobin method with Drabkin’s solution (Spectrum Diagnostics, Obour city Industrial area, Egypt) was used to measure the hemoglobin concentration. Packed cell volume (PCV) was determined using the microhematocrit method. For the differential leukocyte count, blood smears were prepared and examined under a computer-assisted light microscope using a 100× oil immersion lens [27]. According to [28], the mean corpuscular hemoglobin concentration (MCHC) was calculated. To directly quantify the mean corpuscular volume (MCV), an automated Coulter LH 750 hematology analyzer (Beckman Coulter, Fullerton, CA, USA) was utilized [29].

#### 2.5.2. Serum Biochemical Measurements

All biochemical analyses of serum were completed using colorimetric techniques. The total protein (TP) and albumin (ALB) levels in serum were determined by methods described by [30]. Serum globulin was calculated by subtracting the albumin value from the total protein value of the same sample. The liver enzyme activity aspartate aminotransferase (AST), glutamic–pyruvic transaminase (ALT), and alkaline phosphatase (ALP) in serum were measured according to the method described by [31]. The serum complement (C3) levels were assayed using C3 kits as described previously [32], while serum total immunoglobulin (Ig) (mg mL/1) was measured according to [33].

### 2.6. Chemical Analysis of the Fish Samples

Chemical analysis of whole-body fish composition was performed for moisture, crude protein, fat, and ash using fifteen fish at the beginning of the trial and five fish per replicate at the end of the experiment, according to [34].

### 2.7. Total RNA Extraction and cDNA Synthesis

Approximately 50 mg of liver tissue was homogenized in phosphate-buffered saline (PBS) and used for total RNA extraction with Bizol (BioFlux, Japan) according to the manufacturer’s instructions. The integrity of the isolated RNA was verified by electrophoresis on a 2% agarose gel stained with ethidium bromide. Two micrograms of the RNA were used for the complementary DNA synthesis using the ABT 2X RT Mix (cDNA Synthesis Kit, Applied Biotechnology, Ismailia, Egypt) according to the manufacturer’s protocol.

### 2.8. Gene Expression Analysis Using Quantitative Real Time PCR

The qRT PCR reaction was carried out for the expression analysis of *ANFIV*, fat metabolism-related genes (*SREBP1*, *CD36*, and *FAS*), and immune-related genes (*IGMH* and *IL-1B*). In a total volume of 20 µL, consisting of 10 µL of SYBR Green master mix (Applied Biotechnology, Ismailia, Egypt), 1 µL of forward primer (50 nm), 1 µL of reverse primer (50 nm), 2 µL of cDNA, and 6 µL of RNase free water. The program included an initial denaturation at 95 °C for 10 min followed by 40 cycles of 95 °C for 15 s and gene-specific annealing temperatures as presented in Table 3.

### 2.9. Experimental Data Analysis

Using SPSS 23.0, two-way ANOVA was used to examine the impact of two dietary levels of PG or EOs, as well as their potential interactions on the studied parameters. Tukey’s post hoc test was used to determine the significant differences between groups. One-way ANOVA was conducted to examine the combined effect of all levels of the PG or EOs with the control group.

The gene expression was quantified by comparative threshold cycle (ct) method, and the results were expressed as fold change compared to the calibrator after the normalization of the transcript amount to *actb* and *ef-1a* genes as the endogenous control. The relative quantification of target gene expression (*ANFIV*, fat metabolism genes, and immune-related genes) was calculated using the 2^−ΔΔCt^ method according to [38].

## 3. Results

### 3.1. Growth Performance and Feed Utilization

In this study, the growth performance of *O. niloticus* with different levels of PG and EOs is presented in Table 4. Fish reared on both levels of PG showed a significant increase (*p* ≤ 0.05) in FW, DWG, SGR, and RGR compared with the control and both doses of EOs. No significant difference was observed between the two doses of EOs except for SGR where a large dose is significantly higher than a small dose. No significant differences were observed among all groups in IW, WG, SR, and K.

The effect of PG and EOs of feed utilization is shown in Table 5. The best FCR was recorded in fish receiving both feed additives compared with the control; however, only the two doses of PG and high dose of EOs showed significantly improved FCR. Furthermore, the results revealed that dietary supplementation with PG significantly (*p* ≤ 0.05) improved PER, EG, and EU compared with the control group, with no significant differences compared with the EO groups. Nonetheless, for CE, both PG doses followed by the high EO dose were significantly superior to the low EO and the control groups. No significant differences were observed among all groups in FI and PPV.

### 3.2. Chemical Composition of Nile Tilapia

Nile tilapia whole-body composition is presented in Table 6. Moisture content and dry matter remained unaffected by treatments while significant variations (*p* ≤ 0.05) were detected in crude protein (CP), fat, and ash content. The low concentration of EOs significantly (*p* ≤ 0.05) resulted in the highest CP content, and both concentrations of PG significantly (*p* ≤ 0.05) produced the best lipid content. Concerning ash content, PG and EOs significantly reduced ash levels across the different concentrations.

### 3.3. Serum Biochemical Analysis

Serum biochemical analysis was influenced by the different concentrations of PG and EOs as illustrated in Table 7 showing the physiological response of Nile tilapia A significant decrease in glucose levels (*p* ≤ 0.05) was observed at low EO concentrations and at the high PG concentration, while cortisol levels were significantly reduced at low EO concentrations and at both PG concentrations. Both doses of PG and EOs significantly reduced (*p* ≤ 0.05) the level of ALT as compared to the control group. Conversely, the response of AST levels was different, as the two EO doses, as well as the high dose of PG, reduced the levels of AST significantly (*p* ≤ 0.05), whereas the low dose of PG unexpectedly caused a significant increase in AST levels. Low-dose PG and high-dose EOs showed higher values of alkaline phosphatase (ALP) compared with the other doses and the control group.

Albumin significantly (*p* ≤ 0.05) declined at low concentrations of PG and high concentrations of EOs, whereas the high dose of PG and low dose of EOs maintained albumin levels comparable to the control. Total protein (TP) showed significant (*p* ≤ 0.05) reductions in the low-concentration PG and high-concentration EO groups, while the high-concentration PG and low-concentration EO groups showed intermediate TP values that were not significantly different from the control.

Regarding globulin, significant (*p* ≤ 0.05) reductions were observed in both EO concentration groups, while the two concentrations of PG showed intermediate globulin values that were not significantly different from the control.

In the same context, the serum lipid profile exhibited significant alterations where the PG groups showed significantly (*p* ≤ 0.05) reduced cholesterol levels compared to the control. Low-concentration EOs increased the cholesterol level compared to the control. On the other hand, triglycerides (TGs) remained at intermediate levels (131.33 ± 0.67 to 139.67 ± 0.88 mg/dL) in the PG-treated groups; however, there was a significant reduction in both EO groups. HDL was significantly reduced with the high doses of PG and EOs. There was no significant effect on LDL, except with the low PG dose.

The serum immune parameters of Nile tilapia were also influenced by dietary additives, as PG at high concentrations and EOs at low concentrations significantly (*p* ≤ 0.05) elevated C3 levels. However, EOs at high concentrations significantly reduced C3. In the same context, high-concentration PG significantly (*p* ≤ 0.05) enhanced IgM production, whereas both EO concentrations significantly reduced IgM levels.

### 3.4. Hematological Profile

The complete blood count (CBC) analysis is presented in Table 8. The data revealed significant variations in hematological parameters. Hemoglobin (Hb) and red blood cells (RBCs) were significantly reduced with high-concentration PG showing the most serious decline (*p* ≤ 0.05). In contrast, the high-concentration EO group displayed intermediate Hb values. The RBC counts followed the opposite trend, with low-concentration PG showing a significant increase compared to the control. The low concentration of EOs demonstrated the highest values of MCV, MCH, and RDW. Nevertheless, MCHC declined in both low- and high-concentration EO groups.

Total WBCs decreased significantly (*p* ≤ 0.05) in all additive treatments. Lymphocytes remained stable in EO groups but significantly declined in low concentrations of PG. High-concentration PG significantly increased platelet counts, while low-concentration PG significantly increased the monocytic count compared to the control.

### 3.5. Gene Expression

#### 3.5.1. Fat Metabolism-Related Gene Expression Levels

**Figure 1:**
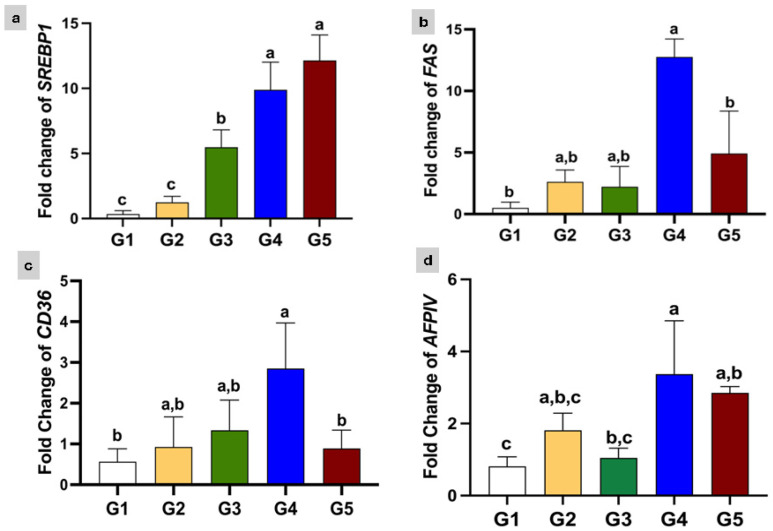
Gene expression levels of (**a**) *SREPB1*, (**b**) *FAS*, (**c**) *CD36*, and (**d**) *AFPIV* genes in liver of Nile tilapia. G1 = control; G2 = PG (5 mg/kg); G3 = PG (7.5 mg/kg); G4 = EOs (1 mg/kg); and G5 = EOs (2 mg/kg). a, b, c: The groups with different letters are significantly different.

The relative expression levels of the *SREBP1*, *FAS*, and *CD36* genes varied significantly among the experimental groups (Figure 1). Regarding the *SREBP1* gene expression, the groups supplemented with EOs (G5 and G4) showed the highest significant expression levels (12.15 ± 1.965 and 9.891 ± 2.127), followed by G3, which received a high concentration of PG (5.484 ± 1.329) (Figure 1a). Similarly, the *FAS* and *CD36* genes showed the highest significant expression in G4 (12.75 ± 1.46 and 2.85 ± 1.118), which received a low concentration of EOs followed by G5 (4.91 ± 03.450 and 0.889 ± 0.45), respectively, as shown in (Figure 1b,c).

The data also indicates the differential expression of *AFPIV* across the experimental groups (Figure 1d). G4, which was supplemented with low concentrations of EOs, showed the most pronounced significant upregulation of *AFPIV* expression (3.372 ± 1.482) followed by G5 (2.853 ± 0.1728), which received a high concentration of EOs, compared to G1. The groups treated with PG at both doses (G2 and G3) also showed an increase in *AFPIV* expression.

#### 3.5.2. Immune-Related Gene Expression Levels

**Figure 2:**
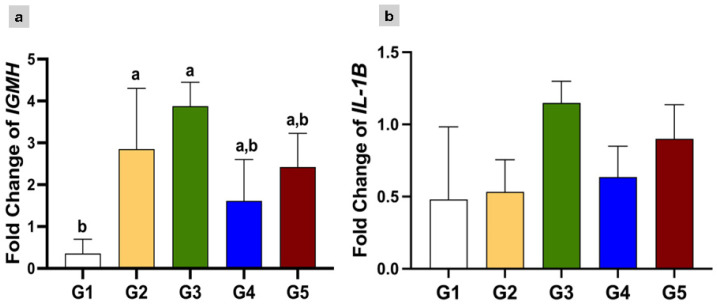
Gene expression levels of (**a**) *IGMH* and (**b**) *IL-1B* genes in liver of Nile tilapia. G1 = control; G2 = PG (5 mg/kg); G3 = PG (7.5 mg/kg); G4 = EOs (1 mg/kg); and G5 = EOs (2 mg/kg). a, b: The groups with different letters are significantly different.

The relative expression levels of the *IGMH* and *IL-1B* genes showed variable patterns among the experimental groups (Figure 2). The groups that received PG at both doses (G2 and G3) showed significant upregulation of *IGMH* expression (1.813 ± 0.4751 and 1.05 ± 0.268) (Figure 2a) relative to the control group (*p* ≤ 0.05). No significant differences were observed among the groups in the expression of *IL-1B* gene (Figure 2b).

## 4. Discussion

In intense aquaculture systems, the health, development, and survival of aquatic species are critically dependent on the quality of the water. All the water quality indicators that were evaluated in this study, such as temperature, pH, dissolved oxygen (DO), and ammonia concentrations, stayed within acceptable bounds and did not significantly differ between the various treatment groups. This consistency suggests that rearing conditions are being effectively managed, which is crucial for reducing stress and fostering *O. niloticus’s* best physiological performance [39,40].

### 4.1. Effect of PG and EOs on Growth Performance and Feed Utilization

In this study, PG showed a statistically significant improvement (*p* ≤ 0.05) in final weight (FW), daily weight gain (DWG), specific growth rate (SGR), and relative growth rate (RGR) compared with all other groups. These findings are in line with recent research demonstrating the potential glycol-based compounds as energy sources to enhance the metabolic efficiency and food absorption of aquatic species [11]. Most growth indices showed no discernible changes between the two EO doses, except for SGR, where the greater EO dose performed better than the lower one, suggesting that EOs have a dose-dependent but constrained ability to stimulate growth. Mehrim et al. [41] observed a similar result when testing graded amounts of caraway essential oil (CEO) in Nile tilapia diets. Their results demonstrated a restricted optimum range for eliciting growth benefits, with tilapia fed 0.1 g CEO per kg of diet achieving considerably better growth performance and feed utilization, whereas greater dosages up to 0.5 g/kg had adverse effects.

Fish groups receiving both PG doses and the higher EO dose had the lowest feed conversion ratio (FCR) in terms of feed consumption. Also, the protein efficiency ratio (PER), energy gain (EG), energy utilization (EU), and carcass energy content (CE) of the PG-fed groups were notably greater than those of the control and EO-fed groups. This indicates that PG has a greater ability to improve protein utilization and energy partitioning in *O. niloticus*, according to Khalasi et al. [11], and this could be because of its rapid metabolic availability and role as a glucogenic substrate.

However, there were no discernible differences in productive protein value (PPV) or feed intake (FI) across any of the groups, indicating that the increases in growth and efficiency measures were due to improved feed use rather than increased feed consumption. Magouz et al. [42] demonstrated that the addition of phytochemicals like PG and EOs can improve feed conversion efficiency without necessarily altering intake or appetite.

The whole-body proximate composition of *O. niloticus* is an essential indicator of its nutritional status and the efficiency of its feed intake. The fish’s moisture and dry matter content in the current investigation were not significantly impacted by PG and EO dietary supplements, suggesting that these additives had no effect on tissue hydration or water balance. Ng and Romano [43] discovered that dietary changes and homeostatic modulation often have little impact on the moisture content of fish.

### 4.2. Effect of PG and EOs on Chemical Composition of Nile Tilapia

There were significant differences in the levels of crude protein (CP), ash, and fat. Remarkably, fish fed diets with low quantities of EOs had the highest CP content. Previous studies have shown that EOs can enhance food absorption and boost the activity of digestive enzymes, which increases the quantity of protein deposited in fish tissues [44].

However, both PG concentrations significantly increased the total body fat content. By increasing gluconeogenesis and consequently modifying lipid metabolic pathways, PG can mitigate unfavorable energy balance in postpartum dairy cows when there is an excess of food energy [44]. Additionally, Ateş et al. [45] found that PG supplementation in Akkaraman lambs improved lipid mobilization and storage by promoting desaturation indices in fat and changing the mix of fatty acids in adipose depots. All these results highlight how PG may be able to preserve protein in fish and mammalian systems while directing metabolic energy toward lipid storage. Aquafeeds looking to optimize growth performance may find this energy-saving function to be quite beneficial. A significant reduction in ash content was observed in treatments receiving both PG and EOs. El-Sayed. [39] suggests that increased growth and tissue accretion may dilute mineral concentrations relative to protein and lipid content.

### 4.3. Effect of PG and EOs on Serum Biochemical Analysis

The serum biochemical parameters of Nile tilapia were significantly affected by food supplementation with varying amounts of PG and EOs. These findings reflect the physiological alterations and stress responses induced by these dietary additives. Notably, fish given a high concentration of PG and a low amount of EOs had significantly lower glucose levels, which may indicate a potential way to mitigate metabolic stress. This is consistent with research on winter-stressed Nile tilapia, which showed that dietary propylene glycol (5–7.5 mL/kg) had glucogenic and stress-buffering properties by dramatically lowering serum glucose and important lipid markers like LDL-C and triglycerides [8].

Serum cortisol (a stress indicator) levels significantly decreased in fish administered low EOs and both PG doses. According to Hoseinifar et al. [46], these reductions may reflect the anxiolytic and antistress properties of EOs and PG through changes in neuroendocrine signaling.

The hepatic enzymes ALT and AST are crucial markers of tissue integrity and liver function. Abdel-Latif et al. [47], found that, the high dose of PG and both EO concentrations significantly decreased ALT and AST levels in this study, indicating potential hepatoprotective advantages. The low dose of PG unexpectedly increased AST, suggesting mild hepatic stress, whereas the non-significant drop in ALP in the high-PG and low-EO groups points to no negative effects on bone or liver metabolism. This is in line with findings in common carp, where dietary thyme essential oil demonstrated a hepatoprotective profile by dramatically lowering AST and maintaining stable ALP activity [48].

Regarding protein metabolism, the groups with low PG and high EOs showed notable decreases in albumin and total protein levels, which most likely indicated altered protein synthesis or early hepatic adjustment. Tilapia fed orange peel essential oil at specific inclusion rates exhibited a comparable drop in serum protein indicators, which was interpreted as a dose-dependent modification of liver-synthesized proteins rather than complete impairment [49].

However, fish that were given high concentrations of PG and low amounts of EOs were able to maintain serum albumin and total protein (TP) levels similar to those of the control group. It is commonly known that albumin and TP are sensitive indicators of fish hepatic function, nutritional status, and protein metabolism. It appears that these food doses did not provide enough oxidative or metabolic stress to interfere with protein synthesis pathways since there were no appreciable changes in these parameters. Since the liver is the main location for albumin synthesis and its damage frequently results in lower amounts of circulating protein, the stability of albumin and TP instead suggests that hepatic viability was maintained [50,51].

Furthermore, sustaining balanced blood protein concentrations during dietary interventions shows a successful approach to nutrient use, suggesting that low-EO and moderate-PG supplementation did not divert amino acids from anabolic activities or place a metabolic strain on the body. Similarly, dietary additives at appropriate amounts maintained normal protein turnover and hepatocellular function in tilapia and other farmed fish species, whereas excessive dosages caused disruptions in serum protein profiles [47,52]. In contrast to the metabolic changes shown at higher EO doses, the preservation of albumin and TP in these groups thus demonstrates a dose-dependent effect of PG and EO supplementation, where modest inclusion maintains hepatic homeostasis and protein metabolism. However, globulin levels in PG-treated groups remained unchanged, indicating that PG had a more neutral or advantageous effect on humoral immunity.

The discovery that low-dose EOs resulted in a modest but surprising increase in plasma cholesterol might be an indication of a dose-dependent biphasic action of certain terpenoid components. Essential oils can occasionally change bile acid turnover or promote hepatic lipid synthesis at modest inclusion rates before any hypolipidemic reaction is noticeable. Similar hermetic patterns were seen in Nile tilapia by [53], where sterol metabolism was regulated but greater lipolysis was not triggered by suboptimal essential oil levels. Such reactions could be mediated by the feedback regulation of HMG-CoA reductase or the partial activation of nuclear receptors (for example, PPARα). Therefore, the effect of low EOs on cholesterol does not necessarily suggest disease; rather, it may represent an adaptive hepatic adjustment that occurs before the lipid-lowering effects observed at higher dosages.

High EOs dramatically decreased triglyceride levels, which stayed within a narrow range, indicating a possible increase in lipid metabolism. Although there is currently little data specific to fish, comparable hypolipidemic effects have been reported in mammals. The possibility of dietary phytogenic substances promoting lipid clearance through increased metabolic turnover is supported by the fact that coriander seed extract significantly reduced the levels of cholesterol and triglycerides in the tissues of rats given a high-fat diet [54].

In contrast, Sosaet et al. [53] discovered that essential oil supplementation in Nile tilapia juveniles greatly boosted HDL concentrations, demonstrating how energy-rich diets can regulate lipoprotein profiles in distinct ways. High dosages of both feed additives drastically decreased HDL cholesterol. LDL levels were largely unaffected, except for a slight decline in the low PG group, which might have been caused by minor changes in lipid absorption or transport dynamics [55].

Finally, immunological indicators including complement component 3 (C3) and immunoglobulin M (IgM) were significantly impacted by dietary treatments. High doses of PG significantly raised both C3 and IgM levels, indicating potential immunostimulatory effects, whether through the activation of innate immune signaling pathways or the improvement in humoral response. When fed a phytogenic feed additive, Nile tilapia showed a similar immunogenic enhancement; the crucial markers of enhanced innate and humoral immune competence, such as lysozyme activity, lysozyme-related immune effectors, and differential blood cell distributions, were significantly elevated [56]. However, IgM levels were dramatically lowered by both EO doses, and C3 was significantly lowered by high EOs. This may suggest that some of the phytochemicals in EOs have immunosuppressive effects that are dose dependent. This pattern is consistent with research on common carp (*Cyprinus carpio*), which showed that adding Origanum vulgare essential oil (OEO) to the diet had dose-dependent antioxidant and immunomodulatory effects. However, low doses of OEO did not improve lipid clearance, pointing to an early phase activated metabolic process [57].

### 4.4. Effect of PG and EOs on Hematological Profile

Dietary supplementation with varying amounts of PG and EOs had a substantial effect on Nile tilapia hematological profile. Fish exposed to high doses of PG showed a considerable drop in Hb and RBC levels, which may indicate hemolytic stress or impaired erythropoiesis at excessive PG levels. Such declines in erythrocytic markers may be a sign of an underlying toxic or oxidative stress effect caused by excessive PG exposure, according to [58].

Fish given high doses of EOs displayed intermediate hemoglobin levels, suggesting a somewhat milder impact on their capacity to carry oxygen. Interestingly, the RBC counts displayed the reverse tendency, rising significantly in comparison to the control group due to the low PG content. Hypoxia studies provide compelling evidence for RBC upregulation as an adaptive response. For instance, within 6 to 24 h of hybrid sturgeon being exposed to lower dissolved oxygen levels, both RBC counts and Hb concentrations increased significantly. These dynamics are consistent with the trend that has been observed, i.e., an elevated RBC count may be caused by mild physiological stress brought on by PG, which stimulates early-stage erythropoiesis [59].

The highest corpuscular volume (MCV), mean corpuscular hemoglobin (MCH), and red cell distribution width (RDW) were seen in fish receiving modest dosages of EOs. Significant alterations in blood biochemical parameters and immune cell profiles were observed in Nile tilapia feed essential oils of ginger and clove basil, indicating that EOs may modulate the hematological system [60]. Despite these increases, the mean corpuscular hemoglobin concentration (MCHC) was significantly lower in both EO-treated groups, suggesting a dilutional effect or altered hemoglobin synthesis.

Total white blood cell (WBC) counts significantly decreased across all treatments (*p* ≤ 0.05), indicating a general leukopenic response that might be a symptom of immunosuppression caused by the additives. Adaptive immune cells, however, responded to EO supplementation with selective stability, as evident from the fact that lymphocyte numbers in EO-treated groups remained unchanged. Conversely, fish given modest amounts of propylene glycol (PG) showed a marked drop in lymphocyte counts, indicating that PG may have mild immunosuppressive effects even at low concentrations. This is consistent with early human immunology studies showing that PG, a common pharmaceutical solvent, can interfere with innate immune functions. Although these effects were seen in mammalian models, their existence highlights the significance of giving careful thought to PG’s immunomodulatory potential in all species [61]. Other animals, including dairy cows during the early stages of lactation, have shown systemic hematological responses to PG supplementation. In these cases, PG influenced blood metabolites and promoted energy balance [62]. Moreover, low PG concentrations led to an increase in monocyte counts, which may be a sign of a low-grade inflammatory reaction or an early immunological response because monocytes often rise in response to non-specific immune stimulation [63].

### 4.5. Effect of PG and EOs on Gene Expression

In the context of dietary supplementation with PG and EOs, *O. niloticus*’s expression of fat metabolism, AFPIV, and immune-related genes demonstrated distinct dose-dependent and treatment-specific responses, indicating systemic metabolic and immunological adjustments that are consistent with the observed serum biochemical and hematological profiling. The elevation of *SREBP1* and *FAS* genes in groups G3, G4, and G5, particularly in EO-supplemented groups, suggests the stimulation of hepatic lipogenesis pathways, according to genes related to fat metabolism [64]. These results are in line with the serum lipid data, which shows that both low and high EO concentrations affect triglyceride and cholesterol levels, with high EO concentrations considerably lowering TG concentrations. The lipid profile changes are consistent with the increased expression of *CD36* in the EO-treated groups, suggesting improved intracellular processing and fatty acid uptake. Similar *CD36*-mediated lipid uptake has been shown in fish and mammals that consume large amounts of unsaturated fats in their diets, where hepatic lipid buildup is accompanied by elevated *CD36* transcription [65,66,67].

However, the high EO-supplemented animals maintained lower serum triglyceride levels even while lipogenic genes including *SREBP1* and *FAS* were upregulated. A feedback regulatory mechanism that balances increased lipid mobilization and β-oxidation with greater lipid synthesis capacity could explain this conundrum. The dynamic interaction between lipogenesis and lipolysis/oxidation determines lipid homeostasis, and increased lipogenic gene expression can be countered by activating lipolytic pathways [64]. It has been demonstrated that components of essential oils, such limonene and eucalyptol, increase mitochondrial activity and fatty acid oxidation, which in turn promotes the removal of triglycerides [68]. Furthermore, lipolysis itself ensures systemic lipid use by acting as a signaling mechanism that triggers the transcription of genes linked to fatty acid oxidation and energy expenditure [69].

Similar results are also seen when thyroid hormone stimulation is applied, when lipid mobilization and oxidation cause lipogenic gene expression to rise while hepatic triglyceride concentration falls [70] Taken together, these findings suggest that EO supplementation may activate two regulatory mechanisms simultaneously: the stimulation of lipogenesis and the enhancement of lipid catabolism. This would account for the observed decrease in serum triglyceride levels despite the increased expression of lipogenic genes. A balanced metabolic adaptation to PG is shown by the modest elevation of lipid-related genes in G3 recipients of high PG doses, which also correlates with the decrease in serum cholesterol in PG-treated groups.

Conversely, the observed elevation of *AFPIV* expression, especially in groups treated with EOs, might be a result of a coordinated hepatic response to environmental and metabolic changes, which could connect *AFPIV* to lipid regulatory pathways. *O. niloticus’s* type IV antifreeze gene was described by [21], who also showed that seasonal temperature variations dynamically alter the gene’s transcription in tandem with changes in several immune-related genes. Their findings provide credence to the idea that *AFPIV* is not just a cryoprotectant but can also be induced in more extensive stress situations, acting in part as an acute-phase-like mediator that may have metabolic and immunological implications. Together with the lipid-active signaling that the essential oils in the current experiment triggered, the temperature-dependent expression patterns they reported imply that *AFPIV* upregulation may be a component of a stress-adaptive hepatic program that affects energy allocation, membrane stability, and potentially lipid transport.

It is interesting to note that, particularly in the G4 group, which received a low dose of EOs, the elevation of *AFPIV* was accompanied by a substantial activation of lipogenic genes such *SREBP1*, *FAS*, and *CD36*. While *CD36* acts as a membrane transporter that mediates fatty acid uptake and processing, *SREBP1* is a master regulator of hepatic lipid metabolism that stimulates the transcription of enzymes involved in fatty acid synthesis, including *FAS* [67].

A coordinated hepatic response to elevated energy demand or membrane remodeling may be reflected in the simultaneous activation of *AFPIV* and lipogenic genes. When lipid synthesis pathways change, *AFPIV*, which shares structural similarities with apolipoprotein A-I, may help with lipid transport or membrane integrity [51]. Furthermore, a hepatoprotective environment that supports such gene regulation is suggested by improvements in antioxidant defenses seen in tilapia treated with EOs, which are characterized by increased catalase activity and decreased lipid peroxidation [52]. Accordingly, decreased serum ALT and AST levels in these groups indicate improved liver function, which is consistent with findings in fish oil-supplemented mice that demonstrate lower hepatic triglycerides and histological indicators of damage [71]. These results suggest that *AFPIV* serves a dual purpose, functioning as a stress biomarker and perhaps modifying lipid homeostasis, especially in liver tissue, which requires more research.

The immunomodulatory function of PG and EOs is further supported by the results on immune-related gene expression. In accordance with increased serum levels of IgM and *C3*, both *IGMH* and *IL-1B* were markedly elevated in PG groups, especially in the high-PG group. This suggests that the humoral and innate immune pathways are being stimulated. Atlantic salmon treated with the heat-killed *Lactiplantibacillus plantarum* strain L-137, a dietary functional feed component, has shown comparable immune-stimulatory patterns. Without affecting growth or the integrity of the microbiota, this additive dramatically raised plasma IgM levels and IL-1β production in the distal intestine in both vitro and vivo settings. This shows that dietary interventions can activate both the innate and adaptive immune systems in aquaculture species [72].

Additionally, the hematological results corroborate molecular information. Low levels of PG increased RBC, Hb, and HCT values, which may have been brought about by better erythropoiesis and oxygen transport. These results were consistent with increased immune gene expression and biochemical indicators of increased metabolic activity. On the other hand, despite moderate expression of immune genes, the EO groups, specifically those at high doses, appeared to display intermediate or lowered hematological indices, which is consistent with the downregulation of immune responses observed at the transcriptional and serum protein levels. Their lower serum levels of IgM and C3 also suggested that EOs at higher doses may downregulate systemic immunity, either through immunosuppression or anti-inflammatory impacts, which comply with the previous findings of phytogenic feed additive investigations [57].

The immunomodulatory and metabolic-enhancing effects of PG, together with the influence of essential oils (EOs) on lipid metabolism, suggest that their combined application could provide synergistic benefits in aquaculture. Optimizing the dosage of these compounds as functional feed additives may enhance growth performance, support metabolic efficiency, and improve overall health outcomes in cultured species.

Bassolé et al. [73] indicate that combining essential oils (EOs) with their isolated constituents is a promising strategy to enhance EO functionality in food systems. Such mixtures exploit synergistic and additive effects, resulting in improved antimicrobial efficacy compared with individual components.

## 5. Conclusions

Overall, propylene glycol can greatly enhance Nile tilapia (*O. niloticus*) growth performance and feed consumption, and the transcriptional activity of fat metabolism and immune genes strongly correlates with biochemical and hematological indices, revealing how PG and EO additives modulate physiological processes in Nile tilapia. PG at higher doses appears to promote immune activation and support metabolic efficiency. Meanwhile, EO supplementation enhances lipid metabolism but may suppress some immune functions at higher concentrations. These findings underscore the importance of dose optimization for functional feed additives to achieve desirable growth and health outcomes in aquaculture.

## Figures and Tables

**Table 1:** Ingredients and chemical composition of basal diet.

Ingredient	%	Chemical Composition	%
Fish meal (60% CP)	3.2	Dry matter	90.0
Corn gluten	8.0	Ether extract	6.02
Yellow corn	12.2	Crude Fiber	4.95
Poultry byproduct meal	4.0	Carbohydrates	53.93
Wheat middling	22.5	Ash	5.1
Rice bran	8.0	Available phosphorus	0.4
Soybean meal	36.5	Crude protein	30.0
Soybean oil	2.0	Calcium	0.99
Monocalcium phosphate	0.6		
Common salt	0.5		
Calcium carbonate	0.5		
* Premix	2.0		

* Premix: vitamin A (3300 IU), vitamin E (200 mg), vitamin B1 (133 mg), vitamin D3 (410 IU), vitamin B2 (580 mg), vitamin B12 (50 mg), biotin (9330 mg), natural betaine (16 mL), vitamin B6 (410 mg), vitamin C (2660 mg), inositol (330 mg), para-aminobenzoic acid (9330 mg), niacin (26.60 mg), pantothenic acid (2000 mg), iron (200 mg), copper (25 mg), iodine, manganese (325 mg), and cobalt (5 mg). NFE: nitrogen-free extract calculated as follows: NFE = 100 − (crude protein + ether extract + crude fiber + ash).

**Table 2:** The effect of PG and EOs on water quality metrices in different groups of Nile tilapia.

Parameter	G1	PG		EOs	
		G2	G3	G4	EO 2
Temp.	26.66 ± 0.47	26.50 ± 0.49	26.53 ± 0.48	26.62 ± 0.50	26.79 ± 0.49
pH	7.65 ± 0.04	7.60 ± 0.05	7.50 ± 0.05	7.53 ± 0.04	7.49 ± 0.05
DO	4.39 ± 0.25	4.38 ± 0.24	4.43 ± 0.25	4.37 ± 0.24	4.34 ± 0.24
NH_4_	0.51 ± 0.12	0.46 ± 0.10	0.51 ± 0.11	0.64 ± 0.15	0.63 ± 0.15
NH_3_	0.01 ± 0.00	0.01 ± 0.00	0.01 ± 0.00	0.01 ± 0.00	0.01 ± 0.00

Temp. = temperature; DO = dissolved oxygen; NH_4_ = ammonium; and NH_3_ = ammonia. Each value represents mean ± SE.

**Table 3:** Primer sequences used in qRT-PCR.

Gene Name Acc. No.	Sequences	Amplicon Size	Ann. Temp	References
***ANFIV*** **(KY318513.1)**	F: CCGCTGCCATCAGCAACTAA R: CCCTGATGTAAGTCAGCCAT	127	60	[21]
***SREBP1*** **(XM_00545777)**	F: TGCAGCAGAGAGACTGTATCCGA R: ACTGCCCTGAATGTGTTCAGACA	102	59	[35]
***FAS*** **(GU433188)**	F: TCATCCAGCAGTTCACTGGCATT R: TGATTAGGTCCACGGCCACA	102	59	[35]
***CD36*** **(XM_003452029)**	F: TGGAGCACTGGACATCAGTTCCT R: CCCAACACAACCTCCCGTAGATAT	96	59	[35]
***IGMH*** **(KJ676389.1)**	F: AGGAGACAGGACTGGAATGC ACAA R: GGAGGCAGTATAGGTATCAT CCTC	117	60	[36]
***IL-1B*** **(XM_003460625.2)**	F: CAAGGATGACGACAAGCCAACC R: AGCGGACAGACATGAGAGTGC	128	60	[37]
***actb*** **(XM_003455949).**	F: TGGCAATGAGAGGTTCCG R: TGTGTGGTGTGTGGTTGTTTTG	136	60	[37]
***ef-1a*** **(KJ123689)**	F: CTACGTGACCATCATTGATGCC R: AACACCAGCAGCAACGATCA	106	60	[35]

**Table 4:** The effect of PG and EOs on the growth performance of Nile tilapia.

Parameter	G1	PG		EOs		*p*-Value		
		G2	G3	G4	G5	Add. *Conc. (Additive vs. Concentration Interaction)	Add. (PG vs. EO)	Conc. (Low vs. High)
**IW (g/fish)**	31.6 ± 0.36	31.9 ± 0.49	31.93 ± 0.12	32.0 ± 0.53	31.17 ± 0.48	0.121	0.245	0.302
**FW (g/fish)**	83.07 ± 2.27 ^b^	102.99 ± 0.34 ^a^	103.12 ± 6.85 ^a^	98.10 ± 3.38 ^ab^	98.80 ± 2.97 ^ab^	0.049	0.071	0.010
**WG (g/fish)**	52.30 ± 3.1	71.09 ± 0.71	71.19 ± 6.95	66.10 ± 2.93	67.64 ± 3.06	0.064	0.086	0.012
**DWG (g/fish/day)**	0.82 ± 0.031 ^b^	1.13 ± 0.011 ^a^	3.13 ± 0.11 ^a^	1.05 ± 0.046 ^ab^	1.07 ± 0.049 ^ab^	0.064	0.186	0.012
**SGR** **(g/fish/day%)**	1.53 ± 0.03 ^b^	1.86 ± 0.03 ^a^	1.85 ± 0.11 ^a^	1.78 ± 0.03 ^ab^	1.83 ± 0.06 ^a^	0.104	0.100	0.016
**RGR**	262.8 ± 4.84 ^b^	322.99 ± 5.23 ^a^	323.07 ± 22.59 ^a^	306.42 ± 6.46 ^ab^	317.20 ± 11.35 ^ab^	0.128	0.143	0.020
**SR**	100	100	96.67 ± 3.33	100	100	0.475	0.770	0.128
**K**	1.56 ± 0.13	1.62 ± 0.11	1.67 ± 0.12	1.45 ± 0.10	1.43 ± 0.04	0.261	0.790	0.140

Values are presented as mean ± SD. Values with different superscript letters within the same row are statistically significant according to one-way ANOVA analysis followed by Tukey’s post hoc test. *p*-values are shown for the main effects (additives: PG and EOs; concentrations: low and high) and their interaction (Add. *Conc.). G1 = control; G2 = PG (5 mg/kg); G3 = PG (7.5 mg/kg); G4 = EOs (1 mg/kg); and G5 = EOs (2 mg/kg). IW = initial weight; FW = final weight; WG = weight gain; DWG = daily weight gain; SGR = specific growth rate; RGR = relative growth rate; SR = survival rate; K = condition factor.

**Table 5:** The effect of PG and EOs on the feed utilization of Nile tilapia.

Parameter	G1	PG		EOs		*p*-Value		
		G2	G3	G4	G5	Add. *Conc. (Additive vs. Concentration Interaction)	Add. (PG vs. EO)	Conc. (Low vs. High)
FI	107.60 ± 0.32	110.80 ± 0.99	110.27 ± 0.26	111.70 ± 1.15	111.67 ± 0.96	0.409	0.031	0.364
FCR	2.10 ± 0.07 ^a^	1.56 ± 0.03 ^b^	1.58 ± 0.14 ^b^	1.69 ± 0.07 ^ab^	1.66 ± 0.14 ^b^	0.041	0.049	0.007
PER	1.59 ± 0.06 ^b^	2.14 ± 0.04 ^a^	2.15 ± 0.21 ^a^	1.98 ± 0.09 ^ab^	2.02 ± 0.10 ^ab^	0.070	0.125	0.013
PPV	24.83 ± 2.21	34.14 ± 1.11	33.35 ± 3.96	32.17 ± 3.50	33.91 ± 1.87	0.350	0.244	0.138
EG	78.40 ± 6.12 ^b^	125.90 ± 3.33 ^a^	123.09 ± 13.34 ^a^	95.65 ± 9.81 ^ab^	113.17 ± 7.76 ^ab^	0.145	0.520	0.011
EU	15.81 ± 1.20 ^b^	24.68 ± 0.64 ^a^	24.23 ± 2.57 ^a^	18.60 ± 1.91 ^ab^	22.03 ± 1.06 ^ab^	0.151	0.613	0.011
CE	545.83 ± 3.12 ^c^	611.07 ± 1.04 ^a^	616.34 ± 3.93 ^a^	557.50 ± 3.12 ^c^	590.63 ± 0.80 ^b^	0.026	0.989	0.000

Values are presented as mean ± SD. Values with different superscript letters within the same row are statistically significant according to one-way ANOVA analysis followed by Tukey’s post hoc test. *p*-values are shown for the main effects (additives: PG and EO; concentrations: low and high) and their interaction (Add. *Conc.). G1 = control; G2 = PG (5 mg/kg); G3 = PG (7.5 mg/kg); G4 = EOs (1 mg/kg); and G5 = EOs (2 mg/kg). FI = feed consumption; FCR = feed conversion ratio; PER = protein efficiency ratio; PPV = protein productive value; EG = energy gain; EU = energy utilization; and CE = carcass energy.

**Table 6:** The effect of PG and EOs on chemical composition of Nile tilapia.

Parameter	G1	PG		Eos		*p*-Value		
		G2	G3	G4	G5	Add. *Conc. (Additive vs. Concentration Interaction)	Add. (PG vs. EO)	Conc. (Low vs. High)
Moisture	72.37 ± 1.19	72.47 ± 0.58	73.21 ± 0.50	73.89 ± 1.05	72.65 ± 0.70	0.660	0.832	0.709
DM	27.63 ± 1.18	27.53 ± 0.58	26.79 ± 0.50	26.11 ± 1.05	27.35 ± 0.70	0.820	0.473	0.234
CP	54.72 ± 0.97 ^c^	56.23 ± 0.29 ^bc^	56.48 ± 0.41 ^bc^	59.85 ± 0.38 ^a^	58.67 ± 0.35 ^ab^	0.878	0.986	0.845
Fat	25.13 ± 0.26 ^c^	31.14 ± 0.24 ^a^	31.55 ± 0.38 ^a^	23.30 ± 0.51 ^d^	27.52 ± 0.29 ^b^	0.652	0.752	0.573
Ash	18.66 ± 0.68 ^a^	12.89 ± 0.21 ^c^	11.94 ± 0.40 ^c^	15.74 ± 0.23 ^b^	12.51 ± 0.54 ^c^	0.458	0.847	0.202

Values are presented as mean ± SD. Values with different superscript letters within the same row are statistically significant according to one-way ANOVA analysis followed by Tukey’s post hoc test. *p*-values are shown for the main effects (additives: PG and EOs; concentrations: low and high) and their interaction (Add. *Conc.). G1 = control; G2 = PG (5 mg/kg); G3 = PG (7.5 mg/kg); G4 = EOs (1 mg/kg); and G5 = EOs (2 mg/kg). DM = dry matter; CP = crude protein.

**Table 7:** The effect of PG and EOs on serum biochemical analysis in different groups of Nile tilapia.

Parameters	G1	PG		EOs		*p*-Value		
		G2	G3	G4	G5	Add. *Conc. (Additive vs. Concentration Interaction)	Add. (PG vs. EO)	Conc. (Low vs. High)
Glucose	188.00 ± 2.65 ^a^	193.67 ± 1.45 ^a^	163.67 ± 1.20 ^c^	125.33 ± 1.45 ^d^	178.33 ± 0.88 ^b^	0.00	0.00	0.00
Cortisol	25.33 ± 0.38 ^a^	21.72 ± 0.38 ^b^	21.39 ± 0.13 ^b^	19.71 ± 0.30 ^c^	24.3 ± 0.26 ^a^	0.000	0.000	0.168
ALT	55.67 ± 1.45 ^a^	49.33 ± 0.88 ^b^	45.00 ± 1.155 ^b^	33.33 ± 0.33 ^c^	21.33 ± 0.67 ^d^	0.003	0.00	0.00
AST	116.67 ± 0.67 ^b^	171.33 ± 0.33 ^a^	95.67 ± 0.33 ^c^	64.67 ± 0.67 ^d^	29.33 ± 0.88 ^e^	0.00	0.00	0.00
ALP	25.67 ± 0.88 ^b^	39.00 ± 1.55 ^a^	27.33 ± 0.88 ^b^	27.66 ± 0.88 ^b^	36.33 ± 0.88 ^a^	0.000	0.143	0.244
Albumin	2.06 ± 0.01 ^ab^	1.99 ± 0.02 ^bc^	2.08 ± 0.02 ^a^	2.07 ± 0.01 ^a^	1.93 ± 0.03 ^c^	0.00	0.141	0.052
TP	3.93 ± 0.09 ^a^	3.40 ± 0.12 ^b^	3.63 ± 0.12 ^ab^	3.47 ± 0.12 ^ab^	3.37 ± 0.88 ^b^	0.152	0.549	0.374
Globulin	1.88 ± 0.09 ^a^	1.52 ± 0.04 ^ab^	1.62 ± 0.02 ^ab^	1.3 ± 0.13 ^b^	1.43 ± 0.06 ^b^	0.699	0.433	0.086
Cholesterol	175.33 ± 1.20 ^ab^	143.33 ± 1.76 ^d^	158.00 ± 1.00 ^c^	179.67 ± 1.76 ^a^	169.67 ± 1.20 ^b^	0.000	0.132	0.000
TG	137.33 ± 0.88 ^a^	139.67 ± 0.88 ^a^	131.33 ± 0.67 ^b^	123.33 ± 1.76 ^c^	107.33 ± 1.20 ^d^	0.007	0.000	0.000
HDL	53.67 ± 0.33 ^a^	53.67 ± 0.88 ^a^	47.67 ± 0.67 ^c^	51.67 ± 0.67 ^a^	44.33 ± 0.33 ^b^	0.304	0.000	0.001
LDL	94.67 ± 0.88 ^a^	81.33 ± 2.33 ^b^	97.33 ± 3.53 ^a^	103.33 ± 1.45 ^a^	103.67 ± 0.67 ^a^	0.003	0.003	0.000
C3	4.43 ± 0.03 ^c^	3.03 ± 0.03 ^d^	6.13 ± 0.03 ^a^	5.70 ± 0.00 ^b^	2.87 ± 0.03 ^e^	0.000	0.001	0.000
IgM	7.07 ± 0.15 ^b^	6.67 ± 0.15 ^b^	8.33 ± 0.26 ^a^	5.79 ± 0.18 ^c^	5.33 ± 4.7 ^c^	0.000	0.008	0.000

Values are presented as mean ± SD. Values with different superscript letters within the same row are statistically significant according to one-way ANOVA analysis followed by Tukey’s post hoc test. *p*-values are shown for the main effects (additives: PG and EOs; concentrations: low and high) and their interaction (Add. *Conc.). G1 = control; G2 = PG (5 mg/kg); G3 = PG (7.5 mg/kg); G4 = EOs (1 mg/kg); and G5 = EOs (2 mg/kg). ALT = alanine aminotransferase; AST = aspartate aminotransferase; TP = total protein; ALP = alkaline phosphatase; TG = triglyceride; HDL = high-density lipoprotein; LDL = low-density lipoprotein; C3 = complement 3; and IgM = immunoglobulin M.

**Table 8:** The effect of PG and EOs on hematological parameters in different groups of Nile tilapia.

Parameters	G1	PG		EOs		*p*-Value		
		G2	G3	G4	G5	Add. *Conc. (Additive vs. Concentration Interaction)	Add. (PG vs. EO)	Conc. (Low vs. High)
**Hb**	11.45 ± 0.05 ^a^	10.00 ± 0.1 ^c^	8.1 ± 0.1 ^e^	8.85 ± 0.05 ^d^	10.7 ± 0.1 ^b^	0.000	0.000	0.777
**RBCs**	2.07 ± 0.01 ^c^	2.41 ± 0.01 ^a^	1.94 ± 0.01 ^d^	2.11 ± 0.00 ^b^	2.06 ± 0.01 ^c^	0.000	0.000	0.000
**Hematocrit**	29.55 ± 0.15 ^d^	34.55 ± 0.05 ^b^	31.2 ± 0.00 ^c^	41.05 ± 0.05 ^a^	34.25 ± 0.05 ^b^	0.000	0.000	0.000
**MCV**	143.15 ± 0.05 ^e^	144.30 ± 0.10 ^d^	162.15 ± 0.05 ^c^	194.10 ± 0.00 ^a^	167.15 ± 0.05 ^b^	0.000	0.000	0.000
**MCH**	48.15 ± 0.05 ^e^	49.15 ± 0.05 ^c^	57.1 ± 0.00 ^b^	58.5.0 ± 0.10 ^a^	48.75 ± 0.05 ^d^	0.000	0.000	0.000
**MCHC**	33.6 ± 0.10 ^c^	34.10 ± 0.00 ^b^	35.15 ± 0.05 ^a^	30.15 ± 0.05 ^d^	29.15 ± 0.05 ^e^	0.000	0.000	0.690
**RDW**	12.85 ± 0.05 ^d^	12.40 ± 0.10 ^e^	13.55 ± 0.05 ^c^	26.55 ± 0.05 ^a^	26.15 ± 0.05 ^b^	0.000	0.000	0.002
**PLAT**	8.5 ± 0.50 ^b^	8.00 ± 0.00 ^b^	14.5 ± 0.50 ^a^	10.00 ± 0.00 ^b^	9.5 ± 0.50 ^b^	0.000	0.012	0.001
**WBCs**	57.35 ± 0.15 ^a^	46.00 ± 1.00 ^b^	43.30 ± 0.30 ^b^	46.45 ± 0.65 ^b^	44.70 ± 0.40 ^b^	0.452	0.173	0.012
**Neuts**	2.00 ± 0.00	2.00 ± 0.00	2.00 ± 0.00	2.00 ± 0.00	2.00 ± 0.00	N/A	N/A	N/A
**Lemphs**	92.00 ± 0.00 ^a^	90.00 ± 0.00 ^b^	91.50 ± 0.50 ^ab^	92.50 ± 0.50 ^a^	92.00 ± 0.00 ^a^	0.025	0.005	0.175
**Monocytes**	5.00 ± 0.00 b	7.00 ± 0.00 ^a^	5.50 ± 0.50 ^ab^	5.50 ± 0.50 a^b^	6.00 ± 0.00 ^ab^	0.025	0.175	0.175

Values are presented as mean ± SD. Values with different superscript letters within the same row are statistically significant according to one-way ANOVA analysis followed by Tukey’s post hoc test. *p*-values are shown for the main effects (additives: PG and EOs; concentrations: low and high) and their interaction (Add. *Conc.). G1 = control; G2 = PG (5 mg/kg); G3 = PG (7.5 mg/kg); G4 = EOs (1 mg/kg); and G5 = EOs (2 mg/kg). Hb = hemoglobin; RBCs = red blood cells; MCV = mean corpus volume; MCH = mean corpuscula hemoglobin; MCHC = mean corpuscula hemoglobin concentration; RDW = red distribution width; PLAT = platelet; WBCs = white blood cells; Neuts = neutrophiles; and Lemphs = lymphocytes. N/A: Not Applicable.

## Data Availability

Data supporting the findings of this study are available from the corresponding author upon reasonable request.

## Abbreviations

The following abbreviations are used in this manuscript:

Abbreviation	Full Form
PG	Propylene Glycol
EOs	Essential Oils
WG	Weight Gain
DWG	Daily Weight Gain
SGR	Specific Growth Rate
FI	Feed Intake
FCR	Feed Conversion Ratio
PER	Protein Efficiency Ratio
PPV	Protein Productive Value
EU	Energy Utilization
Temp	Temperature
DO	Dissolved Oxygen
NH4	Ammonium
NH3	Ammonia
WBCs	White Blood Cells
RBCs	Red Blood Cells
PCV	Packed Cell Volume
MCHC	Mean Corpuscular Hemoglobin Concentration
MCV	Mean Corpuscular Volume
EDTA	Ethylene Diamine Tetra Acetic Acid
qRT-PCR	Quantitative Real-Time Polymerase Chain Reaction
NIOF	National Institute of Oceanography and Fisheries
IACUC	Institutional Animal Care and Use Committee
bp	Base Pair
TP	Total Protein
ALB	Albumin
AST	Aspartate Aminotransferase
ALT	Glutamic–Pyruvic Transaminase
ALP	Alkaline Phosphatase
C3	Serum Complement
*ANFIV*	Anti-freeze gene IV
*SREBP1*	Sterol Regulatory Element-Binding Protein
*CD36*	Receptor Involved in Fatty Acid (FA) Uptake
*FAS*	Fatty Acid Synthase
*IGMH*	Immunoglobulin H
*IL-1B*	Interlukein-1B
USA	United States of America
RNA	Ribonucleic Acid
DNA	Deoxyribonucleic Acid
cDNA	Complementary DNA
